# At the borders of medical reasoning: aetiological and ontological challenges of medically unexplained symptoms

**DOI:** 10.1186/1747-5341-8-11

**Published:** 2013-09-04

**Authors:** Thor Eirik Eriksen, Roger Kerry, Stephen Mumford, Svein Anders Noer Lie, Rani Lill Anjum

**Affiliations:** 1 The Department of Work and Environmental Medicine, University Hospital of North Norway, Tromsø, Norway; 2 Division of Physiotherapy Education, University of Nottingham, Nottingham, UK; 3 Department of Philosophy, University of Nottingham, Nottingham, UK; 4 UMB School of Economics and Business, Norwegian University of Life Sciences, Aas, Norway; 5 Department of Philosophy, University of Tromsø, Tromsø, Norway

**Keywords:** Medically unexplained symptoms, Causation, Epistemology, Phenomenology, Ontology, Philosophy, Dispositions

## Abstract

Medically unexplained symptoms (MUS) remain recalcitrant to the medical profession, proving less suitable for homogenic treatment with respect to their aetiology, taxonomy and diagnosis. While the majority of existing medical research methods are designed for large scale population data and sufficiently homogenous groups, MUS are characterised by their heterogenic and complex nature. As a result, MUS seem to resist medical scrutiny in a way that other conditions do not. This paper approaches the problem of MUS from a philosophical point of view. The aim is to first consider the epistemological problem of MUS in a wider ontological and phenomenological context, particularly in relation to causation. Second, the paper links current medical practice to certain ontological assumptions. Finally, the outlines of an alternative ontology of causation are offered which place characteristic features of MUS, such as genuine complexity, context-sensitivity, holism and medical uniqueness at the centre of any causal set-up, and not only for MUS. This alternative ontology provides a framework in which to better understand complex medical conditions in relation to both their nature and their associated research activity.

## Introduction

Medical professionals and the medical research community are faced with comprehensive challenges relating to what are termed *medically unexplained symptoms* (MUS). The medical professional is presented with diverse and disjointed symptoms and one strategy (among others) aims at translating these into meaningful diagnostic entities. However, the expected patterns or clustering of symptoms frequently do not fit any known or common classification. We are here referring to possible medical conditions which to a certain extent are considered to be resistant to explanation. Which conditions qualify as medically unexplained is itself a subject of controversy, but some that have been commonly labelled as such are chronic fatigue syndrome (CFS), irritable bowel syndrome (IBS), low back pain (LBP) and fibromyalgia (FM). In what follows we prefer to apply the most common term MUS, which here refers to a decisive characteristic; *absence of explanatory pathology*.

MUS represent a major challenge facing public healthcare in European and other industrialised countries. The US National Institute of Health (NIH) identifies MUS as the most common problem in medicine [1]. Given the unexplained character of these conditions, together with the diversity of diagnostic designations and definitions, estimates of prevalence and costs necessarily become advanced guesswork. Nevertheless, the numbers available give us a clear indication that such conditions are common and represent a significant cost to society. The UK Forum for Mental Health in Primary Care estimates that the national annual healthcare costs of MUS exceed £3.1 billion (of a total of £18 billion) [2]. MUS are linked to a 20-50% increase in outpatient costs and a 30% increase in hospitalisation (ibid.). Health authorities in England, such as the NHS confederation’s Mental Health Network, estimates that up to 20% of new primary care GP appointments concern MUS. In 2007 5% of Canadians (1.2 million people) suffered from MUS, including multiple chemical sensitivity, fibromyalgia and chronic pain [3]. In primary care practice in Germany MUS represented 66% of all reported symptoms with the highest rates among women, younger persons, and non-native speakers [4].

The problem of MUS could be interpreted as an empirical matter, to be solved by the medical field doing more of the same work using the same methods they apply to other diseases. On this view, more observation data, randomised controlled trials (RCTs), symptom counts and classification could ultimately lead to a clearer understanding of these conditions. Alternatively, it is suggested by some that MUS show the limitations of evidence based medicine [5-7]. This would mean that the problem of MUS is a symptom of deeper, underlying philosophical issues that need to be resolved. Addressing the medically unexplained as a philosophical challenge, this paper approaches the phenomena of MUS both from an epistemological and an ontological perspective.

The structure of the paper is as follows. First we show how MUS is an epistemological problem for the medical community, involving aetiological and classificatory challenges. After this we take a step back and ask what this phenomenon that we are struggling to explain actually is: what is the *matter*, so to speak, or “den Sachen selbst”? The final sections of the paper are similarly motivated by the idea that the problem of MUS is linked to deeper ontological and conceptual issues. We end by offering the outlines of an alternative ontology which we argue would provide a better foundation for understanding characteristic features of MUS such as multifactorial aetiology, heterogeneity and medical uniqueness. Arguably, these are features that are present in all causal setups and in all medical conditions, although perhaps not to the same degree as in MUS. With this alternative ontology, therefore, we might be able to throw some new light also on other medical conditions, such as cancer or heart disease. This will not be further addressed in this paper, however.

### Medically unexplained symptoms as an epistemological problem

The problem of MUS is linked to a lack of causal explanations, applicable and meaningful diagnostic descriptions and specifically targeted interventions addressing such conditions. This problem can be further articulated in different ways:

the bio-physical *causes* of the symptoms, or different factors in the development of the disorder, are unknown;

some of the supposed factors involved in the development of the disorder are known, but the underlying *mechanisms* are not understood;

no adequate psychological or organic *pathology* can be found.

the symptoms remain *undiagnosed* after medical examination.

A review of the comprehensive literature concerning MUS reveals a general expectation that symptoms and their underlying conditions can ultimately be given some kind of explanation, if there were enough research undertaken. This means that the label “unexplained” only indicates that the causal mechanisms are temporarily hidden and undetected. Symptomatic of this view is Vandvik’s [8] comment on IBS: “While we are waiting for a possible explanation for this and numerous other enigmatic conditions” [p. 661, our translation]. Similarly, Yunus [9] discusses the “meta-diagnosis” of central sensitivity syndromes (CSS) and an appurtenant “lower-level” diagnosis such as chronic fatigue syndrome (CFS) suggesting that: “A variety of abnormal neuroendocrine, immunological, and brain functions have been demonstrated in CFS, but their causal relationship with fatigue remains to be determined” [p. 344]. From the medical perspective the *prima facie* problem of MUS is thus an epistemological one, concerning our incomplete knowledge.

There are a number of characteristic features of MUS that make them particularly difficult to handle scientifically. In the following we will present some of these aspects.

### No single and simple cause

No common cause or set of causes can be found for any of these symptoms. Rather, there seems to be a whole range of both symptoms and causes, none of which offer a clear-cut one-to-one relation between cause and effect. As noted by Voigt et al. [10] “patients with MUS do not appear to have monocausal simplistic somatic explanations for their complaints” (p. 408). Several studies show that patients have multiple explanatory models that are used for grasping the complexity of their own conditions. Such models cover the whole spectrum from physical, psychological, social and existential explanations, neither of them being necessarily dominant [11].

Studies of so-called Somatoform Disorder (SD) (another so-called meta-diagnosis) seem to support this. A study by Hiller et al. [12] showed that the symptom presentation of SD is heterogeneous and therefore contributes to comprehensive complexity. On this evidence they conclude that “the clinical attribution of ambiguous symptoms to a single and simple cause is questionable and not consistent with our current state of knowledge” [p. 10]. Another example of complex and unclear aetiology is low back pain (LBP). Different types of low back pain have been identified and different taxonomies are used to describe type. For example LBP is commonly described as acute or chronic based on the period of time a person has experienced pain. Alternatively, type has been described as specific or non-specific. Non-specific LBP (NSLBP) is of significant concern to health care science due to its complexity and high prevalence. Lifetime prevalence has been reported at up to 84% representing significant costs to society [13]. By definition, NSLBP is not attributable to a known specific cause [14].

Evidence regarding its progression and intervention-response is grounded in uncertainty. Uncertainty regarding all aspects of NSLBP can be accounted for by its seemingly inherent complex nature. However, the persistence of this uncertainty seems at odds with the vast amount of scientific research focussed on the phenomena over several decades. With reference to its aetiology, a brief review of epidemiological science exposes the limits of knowledge of causal responsibility. NSLBP is traditionally thought to be causally related to mechanical stresses on the body created through, for example, posture and lifting which may induce aberrant muscle responses and subsequent pain experiences. However, mechanical factors including lifting, standing, walking, occupational postures, bending, twisting, carrying, and manual handling have been reported as non-causative through systematic epidemiological study [15-20].

Varying degrees of statistical associations have been reported between NSLBP and activity levels, obesity and de-conditioning, but none of these variables can be considered causal [21,22]. The same can be said for factors such as smoking, mood, and hypothesised genetic factors, such as Interleukin-1 gene cluster polymorphisms [23-30].

Structural changes identifiable on imaging have also been considered as causal factors but although some studies demonstrate significant associations between pain and lumbar disc degeneration and disc-space narrowing [31,32], meta-analyses do not support causal claims [33]. Further pathophysiological factors associated with tissue structure and pain mediation, for example nerve growth factor and tumour necrosis factor α, are also weak causal agents [34,35].

In sum, the epidemiological failure to identify causal factors confidently informs the definition and nomenclature of NSLBP, as well as the categorisation of NSLBP as a MUS. It is apparent that NSLBP is a complex phenomenon; further, given the variation of epidemiological responses in different studies and different sub-groups, NSLBP can be considered as highly context-sensitive in terms of any potential causal factor.

High proportions of the population experience NSLBP and associate it with a cause – e.g. bending or lifting. Equally, clinicians listening to and assessing people with NSLBP find it difficult to disassociate the effect from some cause [36,37]. A patient may state, for example, that they bent to lift something and felt a sudden onset of back pain, and since that event they have had back pain. There is a deep intuition here for both the patient and the clinician to consider the lifting event as causal to the pain. Yet this is far from being supported from epidemiological studies. Epidemiologically and aetiologically, NSLBP does not have a cause. In each single case however, it clearly does.

Epidemiology seems to deal well with other complex notions, e.g. hypertension. NSLBP acts however as a more sensitive measure of the scientific limitations of population based methodologies than, say, hypertension. This is not to say that population studies in themselves are limited. It is clear that a massive amount of causal knowledge of health processes has been derived from such studies. So the limitation seems to have something to do with notions outside of the methodologies themselves.

### No single and simple theoretical model

The problem of finding a single cause or set of causes for MUS has led to a revision of the presumed causal understanding, a revision which stresses that the aetiological character of unexplained conditions is multifactorial. That is, the causal patterns involved should include biological, psychological and social factors. The theoretical framework suited for such a complexity is the popular bio-psychosocial model of medicine which was developed as a response to George L. Engel’s pioneering article from 1977 [38], “The need for a new medical model: a challenge for biomedicine”, heavily inspired by general systems theory: a model that insists on the creation of broad-spectrum factorising. Instead of limiting the medical model to specific biological factors (the biomedical model) one has also to include the psyche and the individual in the society: both being dimensions of the human entity that one presumes could be divided into separate elements.

Based on this model, the conditions which are referred to as unexplained somehow rest on a) biological components such as genes, physiological reactivity, immune responses, b) psychological factors such as coping patterns, personality traits, health-related habits, cognition and c) social factors such as social support from family, social and cultural beliefs. However, advocates of Engel’s model, such as Alvarez et al. [39], are sceptical of such an idealised separation of components. They emphasise that the bio-psychosocial model should not at all be interpreted as a theory, a philosophy or a clinical method holding such ideas. Instead they insist that Engel’s approach involved a “humanist look at the patient, and it is not possible to design models that show clinicians how to make clinical decisions in every single case: this concerns essentially something really inherent in the human being: individuality and subjectivity” [p. 179].

Thus, in agreement with Alvarez et al., we find that it is not self-evident that those issues, themes, phenomena or matters involved in MUS allow such factorising and separating initiatives (cf. a-c above). Furthermore, we could suggest that this implicit and unarticulated premise in itself represents one of the main problems for future medical research. Such claims naturally draw us into the prolonged discussion concerning reductionism. Reductionism is the idea that every phenomenon or process can at least in principle be explained or derived from relatively lower level phenomena or processes. This means that causation typically travels bottom up, from micro to macro level. Social phenomena have psychological causes which again can be explained biologically, biochemically, and so on. Reductionism thus promotes a derivative relationship between wholes and their parts, where the nature and behaviour of the whole complex phenomenon is entirely determined by its constituent parts.

Dealing with medically unexplained symptoms, Butler et al. [40] presents in this respect a typical criticism of medicine, referring to the eagerness “to break down complex phenomena in the hope of finding meaning in the simpler constituents (reductionism)” [p. 219]. They add: “Even though the biopsychosocial model emphasizes the importance of understanding the patient’s experience, the philosophical basis is essentially mechanistic” [ibid.]. A more radical departure from reductionism seems necessary for dealing with the genuine aetiological complexity of MUS.

### No clear psyche-soma division

There remains an even deeper challenge here, which is frequently addressed in the MUS literature, namely the assumption of the *psyche-soma* division. In the branches of medicine and psychiatry and to a certain extent even in psychology, one upholds the notion of this functional organisation. That is, one presupposes the possibility of separating and attributing the explaining factors to respectively psyche and soma. By this guiding one is left with different interpretative alternatives: 1) the physical symptoms must be understood as secondary to psychological processes (it is “all in the head”), or; 2) the physical symptoms are primary (that is, “real symptoms”). This unfortunate division is not restricted to the classical biomedical model. Butler et al. [40] argues that even the bio-psychosocial model − which is assumed to integrate the patient’s subjective experiences − also presupposes a mind-body dualism. Such dualism has been challenged by a number of researchers on MUS, but perhaps far more crucial is it that the conditions themselves, such as CFS, seem to resist a clear-cut mind-body division [6].

By separating psychological from somatic mechanisms, the endocrine system from the immunological system, gastrointestinal symptoms from musculoskeletal symptoms, cognitive from social aspects, influence of management from quantitative demands at work, and so on, one assumes that the full picture eventually will emerge. That is, one expects that the nexus of critical mechanisms, causal factors and diverse systems together will create a comprehensible totality. This does not necessarily follow, however.

While the original intention was to have a model that would be better suited to deal with the patient as a unity, one seems stuck within an ontological framework where the world consists of independent mereological parts without any genuine interaction or emergence. As already mentioned, this fits well with the idea of reductionism, to which the bio-psychosocial model was intended as a better alternative. In this context, it seems natural to bring in the well-known allegory of the blind men and the elephant [41]. Each of the blind men were introduced to various parts of an elephant and subsequently asked to describe the character of the animal. Their interpretations differed widely, depending on which part they were investigating. The man studying the foot “saw” a tree, while the one studying the trunk “saw” a snake. We can consider the numerous suggestions for explanatory models, hypotheses and conceptual constructions of the medically unexplained in a similar vein. The gastro-medical specialist “sees” the unexplained irritable bowel. The physical-medical specialist has an eye for the inexplicably painful lumbar region. The psychiatrist “sees” an unexplained mental disorder. In a benevolent perspective we may imagine that the numerous fragments all form part of a scientifically ordered mosaic, which through continued painstaking research and modelling finally may turn into the “true and complete picture” of the phenomenon. In the case of the blind men and the elephant, however, no such coherent picture emerged.

### No clear-cut classification

So far we have been dealing with the aetiological problem of MUS, which is related to the genuine complexity of these conditions. Now we enter a perhaps deeper problem concerning the classification of MUS. In the beginning of this paper we mentioned a number of well-known diagnoses that are often classified as medically unexplained: CFS, LBP, FM, IBS and GAD. But the clear-cut classification and diagnosis is apparent only. Since the symptoms of MUS are typically extremely complex, ambiguous and to a large degree overlapping, none of the conditions are easily classified.

One aspect of this challenge concerns the problematic continuum aspect. By introducing the continuum one realises and acknowledges that complaints, symptoms, affliction or distress to a large extent are dimensional phenomena. That is, they can be located somewhere on a time- and severity continuum. The real challenge is to decide exactly where on this scale the complaints of the patient belong: early phase or close to an endpoint, mild or severe condition, acute or chronic symptoms. One has primarily acknowledged these issues in the face of mental illness and especially when trying to diagnose anxiety and depression. Realising that milder forms of such psychiatric conditions are frequently involved in MUS, it is easy to understand the dilemmas medical professionals are confronted when facing patients with such multi-symptomatic conditions. In view of the seriousness of this challenge, Musalek and Scheibenbogen [42] note that “The problem of inhomogeneous categories and the difficulty of drawing boundaries as well as individual progression of psycho-pathologic phenomena, necessitates a change of paradigm from categorical to dimensional diagnostics” [p. 18].

Accepting such complexity with respect to symptoms, one is seemingly faced with entangled chaos. A number of possible strategies are available to overcome the problem. These strategies can be divided into two categories marked by so-called *lumpers* and *splitters*. The lumpers take the very similar symptom pictures to indicate that “something” is in common and that all functional somatic syndromes are manifestations of a single syndrome [ [43] p. 213]. The wide range of higher-level “meta-diagnoses” is the clearest example of this strategy: bodily distress disorder (BDD) [44], central sensitivity syndrome (CSS) [9], subjective health complaints (SHC) [45], functional somatic syndromes (FSS) [46] and somatoform disorder (SD). In contrast to this, the splitters hold that that the diversity of the conditions show that they are in fact different or unique: “comparable with many diseases with a known pathogenic origin, the symptoms, several non-symptom characteristics, and interventions of somatic syndromes show considerable overlap, but this is in itself insufficient reason to give up separate classifications” [ibid.]. In this disordered landscape, every medical speciality manages its own segment of unexplained special conditions. In a branch such as Occupational and Environmental Medicine one can come across conditions such as sick building syndrome (SBS), multiple chemical sensitivity (MCS) and Electromagnetic Hypersensitivity.

The various diagnoses regarding MUS cannot be regarded as scientifically neutral tools. A number of the diagnoses involve clear aetiological assumptions. This is for instance the case for a diagnosis such as CSS for which Yunus [9] ascribes the following mechanisms: “the CSS concept …is based on mutual associations among the members with overlapping clinical features and are bound by a common pathophysiological glue of central sensitization (CS)” [p. 340]. He emphasises that all labels such as *functional somatic syndromes*, *somatisation disorders*, *psychosomatic syndromes* and *medically unexplained symptoms* actually share a feature; the appearing symptoms are governed by a process of sensitisation. The sensitivity itself is considered to be the clinical manifestation of this process. This sort of theory accommodates considerable aetiological implications which are also considered as extremely problematic. Dealing with the endlessly disputed issue of the psyche-soma division from the outset of psychiatric diagnoses, such as conversion disorder and somatisation disorder, Thomas [47] remarks that: “the DSM definitions of conversion/somatization do not provide anything resembling an operational definition for either one. Without this kind of operational definition, there can be no research capable of establishing causal relationships. In other words, the diagnoses of conversion/somatization have never been validated. The whole argument for causality and indeed the diagnosis of conversion/somatization itself is built on quicksand” [p. 544].

From a critical perspective, we find the higher-level diagnoses unhelpful. The object of study seems to withdraw and the creation of new acronyms could lead to increased confusion rather than clarification. Instead, the widespread use of notions such as *syndrome*, *unexplained*, *distress*, *sensitivity* and *subjectivity* suggests that something in the human nature resists prevailing scientific treatment.

### No common experience

We have seen that existing scientific methods and models fall short in our attempt to understand the medically unexplained conditions. Vast aetiological complexity and lack of clear taxonomy have been presented as scientific challenges. But there is perhaps deeper a problem which relates directly to the shortcomings of existing scientific methods. This is the feature of the medically unexplained symptoms that we refer to as *medical uniqueness*. Each MUS patient seems to have both a unique combination of symptoms and a unique expression of the condition. They are, so to speak, diseased in their own way. Confronted by such a challenge one might wonder whether we should continue this search for the true causal nexus of the unexplained, or whether we are wasting our time on an impossible task.

Although we can trace fragmented evidence in the form of assumed true aetiological factors, we are still left with an incomprehensible and enigmatic “entity” – the suffering human being in its environment. Due to the absence of aetiological clarity, Malterud [48] suggests that when we are confronted with an unexplained complaint such as low back pain we should rethink our strategies: “We can understand muscle-pain in the light of the interaction between body, soul and life conditions. It is not so much to gain by searching for simple explanations and unambiguous findings. Instead we should encourage coping and contribute to a dissolving of those vicious circles maintaining the symptoms” [p. 2356].

At this point it should be obvious that this field represents a challenge that accommodates almost insurmountable aetiological and related obstacles. A brief and extremely simplified review of these reveals several issues that call for further investigation. Although the presentation above in no way relates to a homogenous and clearly defined discourse, what the various contributions have in common is that they all somehow address the multifarious landscape of unexplained illness. Altogether, these different attempts to deal with the medically unexplained reveal a certain degree of bewilderment. This is a problem that neither discredits the medical discipline, nor implies that medical researchers should refrain from further investigations into unexplained matters. However, it possibly indicates that medicine somehow has reached a limit. Thus Deary [5] suggests that MUS are the limit cases of medicine and that they may remain unexplained as long as we maintain the old ontology. Taking this as our starting point for a philosophical reflection, we will now explore the medically unexplained from a phenomenological (existential) perspective.

### The phenomenon of the medically unexplained

The search for a causal explanation of medically unexplained illnesses has involved a clear focus on *why*. In spite of our access to highly advanced scientific methods, how can it be that those mentioned conditions are still unexplained – or at least unclarified? Our preliminary answers may be disturbing. We suggest that MUS and our struggle to understand them indicate that one might have reached the borders of prevailing medical reasoning*.* That is, medicine is confronted by its limit cases. Such “cases” or matters reside from the very beginning outside or beyond the prevailing medical scientific catchments area. If this is a problem that concerns a disease matter, then to search for an answer only to the question of *why* is insufficient. We must therefore return to the basic questions concerning interrogatives such as *what* and *how,* asking ourselves; what are, really, the matters involved concerning the so-called MUS? How do they “appear” or “reside” in the life of humans? Such questions are crucial in an exploration of the ontological foundations of any health related matters involving human beings.

An initial response to such questions − from a phenomenological and existential perspective − indicates that we may be facing complex, but also simple and common, human phenomena. Despite the difficulties concerning deciphering and understanding such phenomena, they somehow relate to the non-complicated sphere of everyday life. Our struggle for linguistic control of such phenomena, with the help of advanced medical terminology, necessarily gets out of hand and the result is a messy world of acronyms. All disease are intertwined with the “the human condition” and the MUS-acronyms seem to be only blind gestures to the world that we as human beings are living in. To say that it is unexplained is a category mistake.

Suggesting that the object of study may not be a bio-medically or a bio-psychosocially constituted entity, our approach relates to that of the anthropologist Gilles Bibeau [49]. His worry is that questions such as “What is human in humans?” and “What is the nature of human nature?” to a large extent will be answered by geneticists, neurologists, artificial intelligence researchers, techno-scientists and owners of biotech companies [p. 355]. In contrast, he notes, the lives of humans involve and are shaped by history, language, meaning, symbolic systems, experience, consciousness and emotions that together form a unity that only to a very limited extent can be rationally explained or described by science [ibid]. These are dimensions representing foreign “objects” in a medicalised and technified world.

Thus, humans qua humans exist in − or face − the “external” world on a level that is specifically human. The difficulty with the human level is that there is no agreement on what humans are, or whether we can even describe humans by referring to human properties. This however does not imply that human diseases should be treated and explained on a lower level, even though many symptoms are expressed on a lower level. Instead, we need to keep our attention on the level at which humans live their lives. The appropriate level for describing human experience and phenomena, we argue, is from a phenomenological perspective; *humans qua humans*.

From such a phenomenological perspective, as humans we do not simply have a sickness; we also have an advanced capability of interpretation. We relate to a sickness in certain ways depending on how we interpret it. We do not simply have the property of being male, strong, depressed or eager. We also *relate* to those properties: we *are* male, strong, depressed or eager in specific ways. And it’s these ways that may be important when we want to access the realm of how different MUS come about.

Addressing this subject further, Eriksen et al. [41] emphasise how predominant symptoms such as fatigue and pain, together with certain biographical dimensions, direct us towards what we consider to be inescapable and fundamental conditions in the life of the imbalanced and distressed modern human being. Phenomena such as Fatigued-Being and Painful-Being are dimensions of life that are considered to be indelible, inevitable and to a certain degree indispensable. Such phenomena could be seen as elements in an aesthetics of resistance [ibid.]. Furthermore, they could be seen as encumbrances that follow the destiny of being a living human being. These are dimensions that are not easily reachable for traditional explanatory advances. (For instance, this will forever be the case as regards the phenomenon we name anxiety, which usually follows incomprehensible pain conditions). At least they are not reachable within the existing paradigm for explanation in medicine.

Following such a phenomenological (and ontological) inspired trail of thoughts, it comes naturally to suggest that questions concerning causation are not the only crucial momentum involved. That is, one recognises that the matters – den Sachen – are basic phenomena that precede a second-order conception of an object, grasped as some multifactorial “thing”. However, this lack of faith in explanatory endeavours, organised by medical research regarding unexplained matters, does not imply a universal rejection of causal concerns as such. Rather, it voices a doubt with regard to the factorising, dissection or reduction that follows from efforts aimed at revealing the mechanics involved. Matters such as fatigue, pain, anxiety and melancholy resist such fragmentation, and for this reason we should be open for research initiatives that somehow accepts holism and at the same time is able to problematise the basic elements of the medical scientific paradigm. Consequently, there is a need for a new initiative that carries explanatory potential, but which is able to accommodate real world complexity.

As we continue to introduce such an initiative, we search for a way to accept humans *qua* humans (and the phenomenological descriptions given), while at the same time trying to re-address the question of causality. The aim is to get a better understanding of medically unexplained symptoms as “natural” (individual) reactions (resistance), and also of how such reactions are instantiated in processes taking place at lower levels.

### Ontology revealed

We have seen that the problem of MUS is more than just a question of finding the true causes of a disease. *Prima facie* challenges are related to the complex nature of these conditions with respect to causes, symptoms, diagnosis and classification. MUS researchers have tried to deal with this complexity by challenging monocausality, reductionism and dualism. The mechanistic and biomedical model has been replaced with the bio-psychosocial model, and one has attempted to re-classify and re-organise the conditions. The aim is to find a way to deal with features that are characteristic of MUS but which many existing methods fail to embrace: multifactorial causation (complexity), extreme heterogeneity (context-sensitivity), medical uniqueness (singularity) and health and disease as belonging to the person as a whole (anti-reductionism). Such issues touch upon our deepest ontological assumptions and cannot be separated from our scientific models, concepts or methods.

The existing ontology might not be one consistent world-view. But from the practice of medicine we can derive a number of ontological assumptions. The search for biomedical causes and treatments of psychological and social phenomena reveals a commitment to reductionism, for instance, and professional divisions of medical specialisms suggest that the various parts and dimensions of human health can be treated as relatively separate and distinct processes. This fits well with an ontology that takes wholes to be the sum of mereological parts which − although they are parts of the same mechanisms − don’t have any genuine interaction.

Other ontological assumptions can be derived from medical methods, such as the use of RCT and observation data. Kerry et al. [50] argue that these methods make clear commitments to a notion of causation that is tightly linked to robust correlations (regularity theory). A basic assumption here is that same cause will give same effect, or at least that similar cause will give similar effect. This idea has recently been challenged [51]. A further ontological assumption, that can be seen from the use of population data, is a commitment to general facts over particular or singular facts. This fits well with the covering law ontology, according to which the particular cases − being similar in all the causally relevant aspects − can be logically derived from a general causal claim. In this model, any context-sensitivity or individual differences are ruled out either by definition or through idealisation. What is left is some idealised or statistically average situation which is supposed to apply to any individual case.

When we cannot find perfect correlations between interventions and effect in the population data, we can make probabilistic conclusions instead based on the statistical findings. Such an inference reveals a frequentist commitment to probability theory. This forces us to commit to genuinely chancy situations where all we can say is that a patient will have a certain probability of getting an effect from the intervention, and where it is impossible to say anything more about why some patients had an effect while others didn’t. The frequentist theory contrasts with propensity theory, which takes probability to be based in individual propensities rather than on a given sequence of events. One might then argue that one patient has a higher propensity of getting an effect from the intervention, based on what we know about their medical history, diet, lifestyle, and so on. In contrast to a probabilistic interpretation, one might want to argue that any result that is less than perfect regularities come down to an all or nothing situation, where predicting the effect of intervention is just a matter of finding the right sub-group. If 3 of 10 patients had an effect, it might be that the larger part of the patients had no chance of effect while three of them had a chance of 1. We see here that the different ways to interpret the statistical result give us different descriptions of reality. These are the ontological and conceptual assumptions that we bring to our methods and our data.

Given the heterogeneity of MUS we can see how population data will have little relevance. Individual variation is the rule rather than the exception, and similar causes seem to give vastly different effect. Where one person gets a chronic back pain from bending down or lifting something, others bend and lift every day without getting back pain.

We see that the old ontology cannot be replaced by anything other than a new ontology. If we try to change bits and pieces, there is a fair chance that assumptions from the old ontology follow into our new approach together with our concepts, methods or models. What we offer next is an ontology that challenges the old one in a number of respects. While MUS seem to be the limit cases of the old ontology, they are exemplary cases within the new ontology.

### Dispositionalism – an alternative ontology

The dispositional ontology is one that arguably best accommodates the features we feel are needed for dealing with MUS: singularism, complexity, holism, heterogeneity, scalarity and emergence. This ontology has its roots in Aristotelian metaphysics but it has been appropriated and modernised in recent decades by the likes of Harré and Madden [52], Mumford [53], Molnar [54], Bird [55] and Mumford and Anjum [51]. The core commitment is to the reality of individual powers or dispositions. On one version of this view all things behave the way they do, not because of external laws, but because of their own intrinsic properties [56].

Typical for dispositions is that they can exist unmanifested. A woman can be fertile without ever getting pregnant and one can have a genetic predisposition for a disease without ever developing it. Some call dispositions causal powers. When a sugar cube dissolves, for instance, it is because it has a real causal power of solubility that is “released” when it meets the appropriate mutual manifestation partner, water. Taken in isolation, a disposition might not even do any causal work. Only through interaction with other dispositions will a causal process be initiated. Furthermore, a disposition can contribute to bring about a number of effects. What effect a disposition contributes to produce will therefore depend on the causal context. Heat, for instance, can causally produce a burn, boiling, steam, melting, explosion, drought, fire, growth, health, death, and many other effects, depending on the manifestation partners.

This model seems particularly apt for medical cases and for cases of MUS in particular. A single causal factor can have a vast number of possible manifestation partners. Which effect it contributes to produce will depend entirely on what context it appears in. A virus has the power to cause an infection, but whether it will succeed in doing so will depend on the other causal powers involved. Some people have a better immune system, for instance, but even this is entirely dependent on context: in periods of stress we might have a weaker immune system. Whether we are infected by a virus also depends on the type of virus, its intensity, vaccines, genetic dispositions, and so on. It should therefore be no surprise to us that two people can have vastly different effects from being exposed to a virus.

Causation is central in medicine, since its ultimate aim is health promotion and disease prevention. Promotion and prevention are both causal notions. On the dispositionalist account, this means that while there is no guarantee of successful outcomes in health science, there can be distinct and sometimes strong tendencies. We can try to prevent disease by causally counteracting or interfering with it. Mumford and Anjum [51] distinguish between two types of causal interference: subtractive and additive. The first strategy is to remove one or more of the causes disposing towards the unwanted outcome; the second is to add something that disposes away from the outcome. An example of subtractive interference would be when someone gives up smoking to counteract hypertension, while taking beta-blockers is a case of additive interference towards the same goal. In the latter case the patients could in principle continue with their unhealthy lifestyle, but in addition take a kind of antidote to counteract an effect of that lifestyle.

We saw that the problem of MUS has been linked to dualism and reductionism, where focus has been directed towards the psyche-soma division, favoring simple physiological causes over complex psychosocial ones. The bio-psychosocial model suggests that health is related to more than just the physiological level and should thus be treated as a more complex matter. But how does this work in theory? A reductionist ontology will take for granted that causation travels bottom up. This means that it is assumed that it is possible to causally counteract an outcome on a macro level by interfering with a causal process on the micro level. Reductionism is thus the idea that the causally efficacious level is the micro-level. This is the ontological view of neuropsychology, genetic determinism and sociobiology, and it is one that is gaining popularity also outside the realm of science.

Dispositionalism favours holism over reductionism. Philosophical holism is the view that on each new higher level there can be some causal autonomy. It might for instance be argued that it does not even make sense to ascribe choices, desires or any intentional properties to genes or neurons, but only to agents. On this view desires, intentions and preferences are properties that belong to a higher level than physiology and biology. While the subject of biology is the organism, the subject of psychology is a person. The philosophically holist idea is that the whole is more than just the sum of its parts. Holism is tightly linked to genuine emergence.

The bio-psychosocial model is not a genuinely holist one, since it treats these dimensions as three separable factors that make a contribution to the illness of an individual. A plausible reason for this is that the medical model is restricted by medical methods, such as RCTs, where the factors that we test for are treated as if they are discrete and separable. Dispositionalism acknowledges, however, that causal powers interact in such a way that all factors compose into what we could call a resultant power of the individual overall. Such composition need not be simply linear. It is not as if we can just add powers and get their sum. Most, if not all, causal production seems to happen through nonlinear composition. This means that when the various manifestation partners come together in the appropriate way, they will start to interact, influence and change each other, producing something different from what each could have produced on their own. It could for instance be that what is produced is a novel phenomenon with an entirely new set of properties, none of which are found in its components. Life, mind, society and justice might all be taken as genuine emergent phenomena, which have causal powers that are specific to their level. This form of dispositionalism thus allows an emergentist perspective in which neither the parts of a person, nor their causal powers, are treated as distinct, separable nor even retaining a distinct identity in the whole. RCTs look for causal factors (e.g. what causes health improvement) one at a time, but according to our holist perspective, we cannot guarantee that those factors will behave the same way in all contexts, such as in different patient subjects with their different and no doubt unique combination of qualities.

Some form of philosophical holism seems essential in medicine, and especially for medically unexplained conditions, which clearly do not only belong to any one particular part of a person but to persons as a whole, in their environment. Such an idea is philosophically compelling. We shouldn’t say that it is eyes that see, nor brains that think, for instance. Seeing and thinking are capabilities of whole persons. An isolated eye could do very little. Similarly, we shouldn’t think of NSLBP as a property only of a patients back, but that it is something belonging to the whole person. And even then, we need not limit the illness to just the confines of the person’s body. As described in relation to the phenomenological perspective, the person can include the whole worldview of the subject, including the context within which they are situated. In that case, treatment should also be at the level of the patient, treating them as a whole instead of attempting to treat one isolated part or function of their body.

Emergence is essential in this context, because it allows for genuine complexity. To move from monocausality to multifactorial causation does not in itself guarantee that we take the complexity seriously. If our methods are designed to treat each factor separately, the phenomenon as a whole is lost even if we include many factors and add them up. Complexity is a core idea of dispositionalism, and this is particularly clear in causation. All actual effects will be multifactorial. The flammability of a match is not alone sufficient for it to light when struck. It will also require the presence of oxygen and reasonably arid conditions. Given that all such factors contribute, and all such may be hypersensitive in relation to what they manifest, then the medical uniqueness of each patient starts to look a credible possibility.

Understanding causal interaction is not only about taking into account all the factors involved and how they compose. It is also a question of magnitude or degree. On dispositionalism causes and effects come in degrees. They are not a matter of “all or nothing”. An open fire has the causal power to warm a room to a high degree whereas a light bulb has it to a small degree. And the effect, of a room being warm, is clearly something that comes on a scale. Similarly, we shouldn’t just think of patients as being ill or not, healthy or not. Symptoms as well as causes come in degrees. Whether a person is ill or not cannot be determined solely from the type of symptoms, but must be considered in a wider context. A small causal factor can be the contribution that tips a causal situation over a threshold, for instance. What effect something will have, is thus entirely dependent on what else is already there in the situation. If a situation is already at a tipping point, it takes very little to get a threshold effect. Flu, while painful enough for someone with a strong general health, is still fairly harmless, but it can kill an old person or someone with a weak immune system. Another example is allergy, where a single peanut can cause great harm for one person while being perfectly fine and nutritious for another. This shows the extreme context-sensitivity of causation. Same cause can have vastly different outcomes. The heterogeneity of MUS should therefore not surprise us, at least not from a philosophical perspective. On the contrary: looking for one single feature that is correlated with a type of MUS appears to be a hopeless oversimplification.

Any such medical uniqueness of the individual is likely to be masked in the methods of RCTs, which deal with statistical averages of sufficiently homogenous groups. The problem is that it is possible that no one be average. We mentioned earlier propensity theory which takes probability to be a matter of individual propensities. Dispositionalism favours such a theory, which means that one would not be inclined to draw conclusions about individual propensities *solely* from a certain statistical distribution. What we need to establish instead is what powers are at work behind *certain* forms of disease, both in the person and in the environment.

## Conclusion

The heterogeneity of MUS becomes easier to deal with from a dispositionalist perspective. The dispositions ontology allows a revised reading of data from population studies, as well as facilitating a meaningful appreciation of cause related to single-instance cases. Returning to data on NSLBP, all above quoted population studies reported some statistical correlations, but nothing strong enough to support causal claims, in an epidemiological sense. Likewise, existing comparison studies do not expose causation between hypothesised factors and occurrence of pain. However, dispositionally, it is the few cases in which an occurrence did happen that are most revealing. The individual cases in which mechanical stress did result in pain are informative about the causal compounds of NSLBP.

Dispositionalism allows us to embrace the characteristic features of MUS: causal complexity, individual variety, context-sensitivity and real emergence. It also allows for a more person centred medicine. Rather than treating illness as a biochemical phenomenon belonging to a part of the organism, it should be considered as a more complex phenomenon that is a part of the human being in a psychosocial context. Dispositionalism also reveals the importance of tailoring a treatment to the patient by looking at their total situation. Exposure to a treatment can then make the situation worse rather than better. Each patient will meet the treatment with a whole set of causal factors from their lifestyle, diet, biology and medical history. Since no two individuals are the same, using the same treatment on two different individuals will in effect be two different treatments. Epidemiological invariance becomes both an impossible and redundant quest for MUS. Only by theoretical abstraction can we be tempted to think that there exists some individual that fits the norm of a statistical average. But a robust ontological foundation to back up such a unity is absent.

On a dispositionalist ontology it should not be expected that there is some average, normal or standard way to express a disease, simply because this average, norm or standard does not exist other than as a methodological derivation and abstraction from a vast amount of correlation-data. Limit cases are often the clearest symptoms we get of something being fundamentally wrong with the theoretical framework. So if we take dispositionalism seriously, this is not something that is specifically related to so-called MUS, but applies also to illnesses that we typically take to be medically explained, such as heart disease. The ontological framework presented here suggests a different methodological approach for dealing with MUS, namely one that favours individually based investigation and treatment to statistical and systematic approaches [50]. What we need, then, is some new tools for dealing with causal singularity, complexity, diversity and medical uniqueness.

We are not arguing that the dispositions ontology and the dispositional theory of causation will help us solve the problems of MUS. Instead we have offered a philosophical framework that takes certain characteristic features of MUS to be essential to causation rather than as problematic limit-cases.

## Competing interests

The authors declare that they have no competing interests.

## Authors’ contributions

TEE was the lead author and RLA led the project. All authors contributed equally to the drafting and re-drafting of the paper, as well as development of ideas and concepts on which the paper is based. The authors' contributions are related to their respective areas of current research and expertise. All authors read and approved the final manuscript.

## Authors’ information

TEE, Cand. Polit is a Special Consultant and Social Scientist at the Department of Work and Environmental Medicine University Hospital of North Norway, Tromsø, Norway and works on medically unexplained symptoms and phenomenology.

RK, MSc FMACP MCSP is Associate Professor in the Faculty of Medicine and Health Sciences, University of Nottingham, and works in collaboration with the University’s Department of Philosophy on the nature of causation in health research and evidence-based medicine.

SANL, PhD is Associate Professor, at Department of Philosophy, University of Tromsø, Norway and works on phenomenology and dispositions.

SM, PhD is Professor at the Department of Philosophy, University of Nottingham, Nottingham, UK and Visiting Professor at the Department of Economics and Resource Management, Norwegian University of Life Sciences, Ăs, Norway.

RLA, Dr. Art is research fellow at the Department of Economics and Resource Management, Norwegian University of Life Sciences, Ås, Norway. SM and RLA work on metaphysics, dispositions and causation.
